# Leiomyosarcoma of the Cephalic Vein

**DOI:** 10.1080/13577140120048917

**Published:** 2001-03

**Authors:** Kieran P. Jefferson, John H. Dixon

**Affiliations:** 1 Department of Urology Bristol Royal Infirmary Bristol UK; 2 Department of Orthopaedics Weston General Hospital Weston-Super-Mare BS23 4TQ UK; 3 FRCS 49 Linden Road Westbury Park Bristol BS6 7RW UK

## Abstract

A 78-year-old man presented with a mass on his right forearm. A 5 x 4 x 3 cm^3^ mass was excised *en bloc* with extensions along the course of the cephalic vein and its tributaries. Histological analysis revealed the mass to be a high-grade leiomyosarcoma arising within the cephalic vein. The tumour was controlled locally and distally until the patient died 10 months later, from an unrelated illness. This is the first reported case of a venous leiomyosarcoma of the cephalic vein.


*Sarcoma* (2001) **5**, 27–30

CASE REPORT

### Leiomyosarcoma of the cephalic vein

KIERAN P. JEFFERSON[1] & JOHN H. DIXON[2]

[1]*Department of Urology, Bristol Royal Infirmary, Bristol, UK, and* [2]*Department of Orthopaedics, Weston General Hospital, Weston-Super-Mare, BS23 4TQ, UK*

**Abstract**

A 78-year-old man presented with a mass on his right forearm. A 5 x 4 x 3 $cm^3$ mass was excised *en bloc* with extensions along the course of the cephalic vein and its tributaries. Histological analysis revealed the mass to be a high-grade leiomyosarcoma arising within the cephalic vein. The tumour was controlled locally and distally until the patient died 10 months later, from an unrelated illness. This is the first reported case of a venous leiomyosarcoma of the cephalic vein.

**Key words:** *venous leiomyosarcoma*

### Introduction

Primary leiomyosarcomas of veins are rare in any site.[1,2] Most arise in the inferior vena cava (more than 120 cases reported) and other large central vessels. This case report describes the first reported case of leiomyosarcoma of the cephalic vein. An extensive literature search revealed only 34 cases of primary venous leiomyosarcoma of the extremities, the vast majority in the proximal veins of the lower limb. We have identified only three other case reports of such tumours in the upper limb.[2–4]

### Case report

A 78-year-old Caucasian male presented with an 18-month history of a painful lump on the dorsum of his right forearm (Fig. 1). He believed that this had originated at the site of a previous intravenous cannula and it had therefore been treated by his general practitioner with antibiotics and non-steroidal anti-inflammatory drugs. The lump had not resolved; indeed, three further lumps had appeared more proximally in the course of the cephalic vein. The patient was being treated for chronic obstructive airways disease and had a history of diabetes mellitus and hypertension. There was no family history of any cutaneous syndrome or malignancy.

On examination, a 3 x 2 x 2 $cm^3$ lump was palpable deep to the skin in the course of the cephalic vein. Three smaller lumps were palpable below the elbow. No other skin lesions were found and there was no palpable axillary or antecubital lymphadenopathy. There were no systemic manifestations evident. A pre-operative chest radiograph was normal.

The mass was excised *en bloc* under tourniquet control with a wide margin and primary wound closure. It was rubbery, arising from the cephalic vein and extended continuously along the vein for 20 cm, branching into tributaries. Vascular reconstruction was not performed. Samples were sent for histology and culture.

A firm, grey–pink tissue mass measuring 55 x 45 x 35 $mm^3$ with tubular extensions at several points was described on macroscopic examination (Fig. 2). The total weight was 55 g. Histology revealed a vascular leiomyosarcoma arising from the wall of the vein and extending intraluminally away from the tumour. The tumour was composed of interlacing smooth muscle fibres with foci of necrosis and areas of hyalinisation. The smooth muscle nuclei were enlarged, multiple, pleomorphic and often bizarre. Mitotic count varied between 5 and 13 per 10 high-power fields (Fig. 3).

Post-operatively, the wound healed well with normal hand and arm function. The patient received adjuvant radiotherapy to the tumour bed (55 Gy administered in 20 fractions over 4 weeks) and, at 3-month follow-up, there was no evidence of local recurrence. Ten months after presentation, the patient died of a myocardial infarction; autopsy examination revealed no evidence of local recurrence or metastasis.

Correspondence to: Kieran P. Jefferson, MA, FRCS, 49 Linden Road, Westbury Park, Bristol BS6 7RW, UK. Tel: (+44) 117-928-3509; E-mail: jeffersons@doctors.org.uk

1357–714X print/1369–1643 online/01/010027–04 © 2001 Taylor & Francis Ltd
DOI: 10.1080/13577140120048917

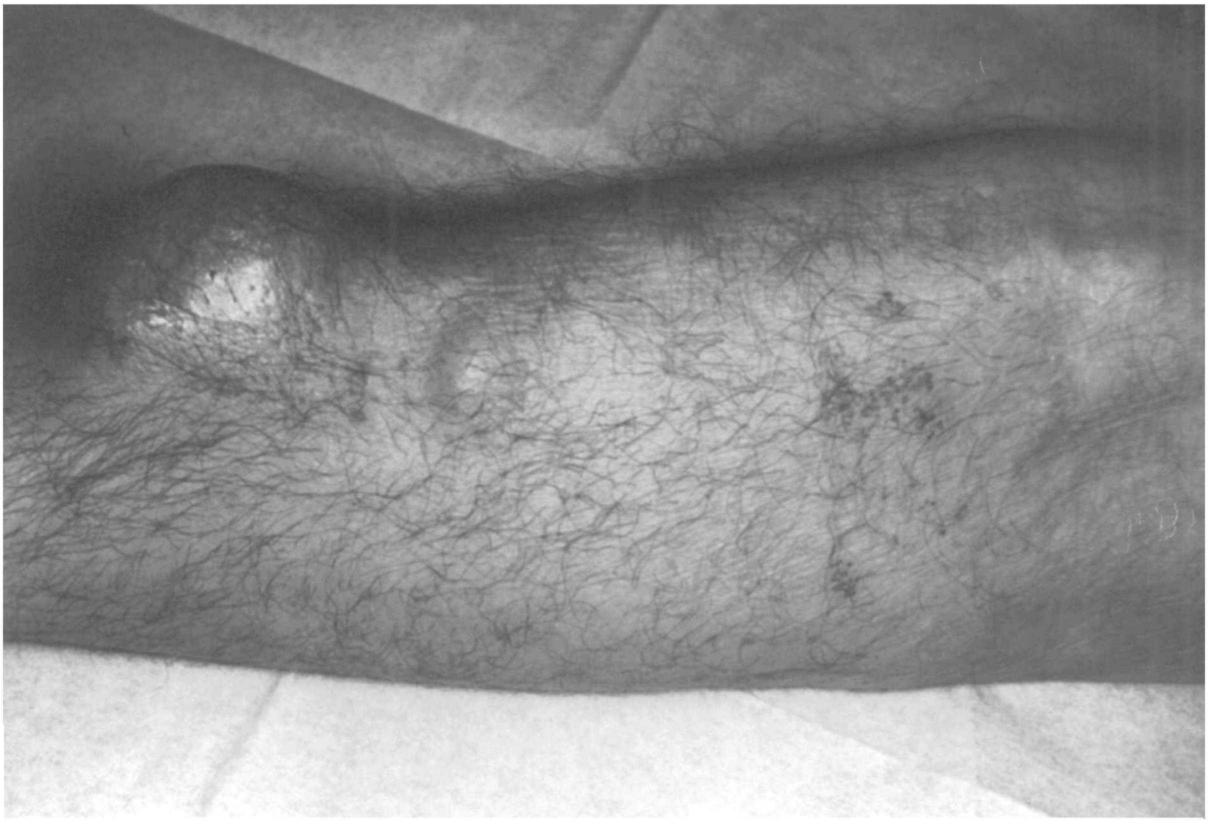

**Fig. 1.** *Appearance of the tumour at presentation.*

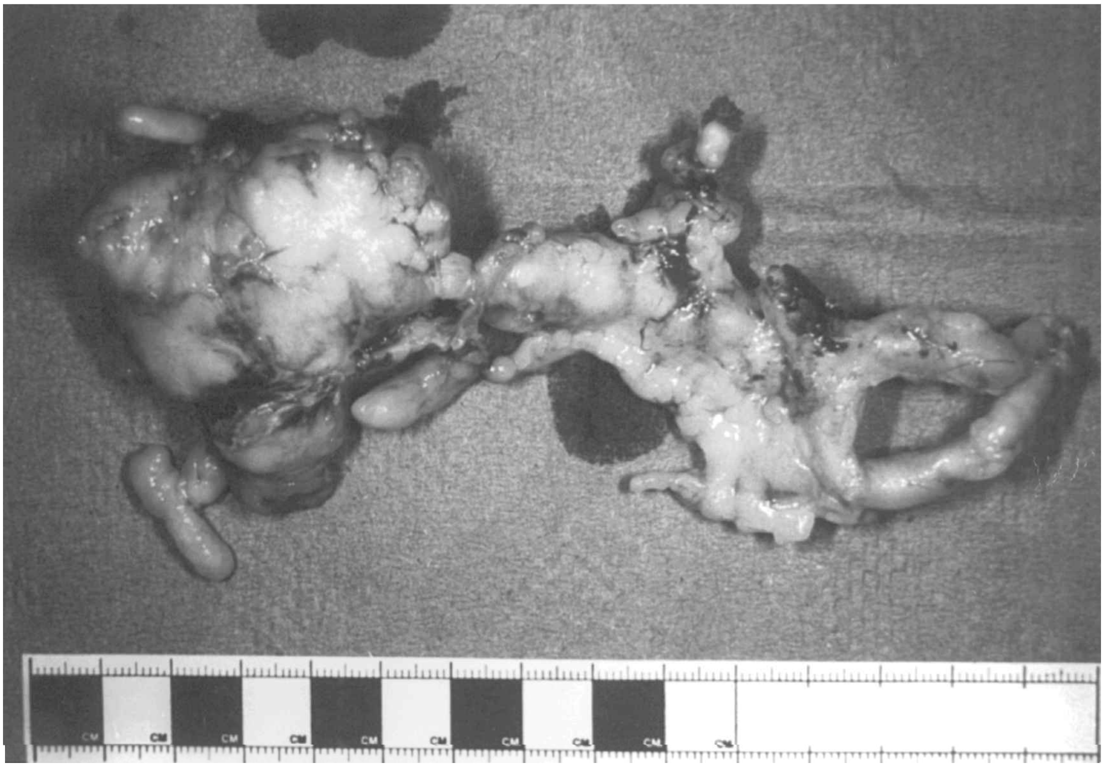

**Fig. 2.** *Macroscopic appearance of the tumour after excision.*

### Discussion

Table 1 contains details of previously reported venous leiomyosarcomas of the extremities. They most commonly present as slow-growing, painless masses; pain is sometimes experienced in association with intraluminal extension of the tumour. Acute venous obstruction is rare due to their slow growth rate.[3,5] Peak incidence occurs in the sixth and seventh decades, and they are similarly more

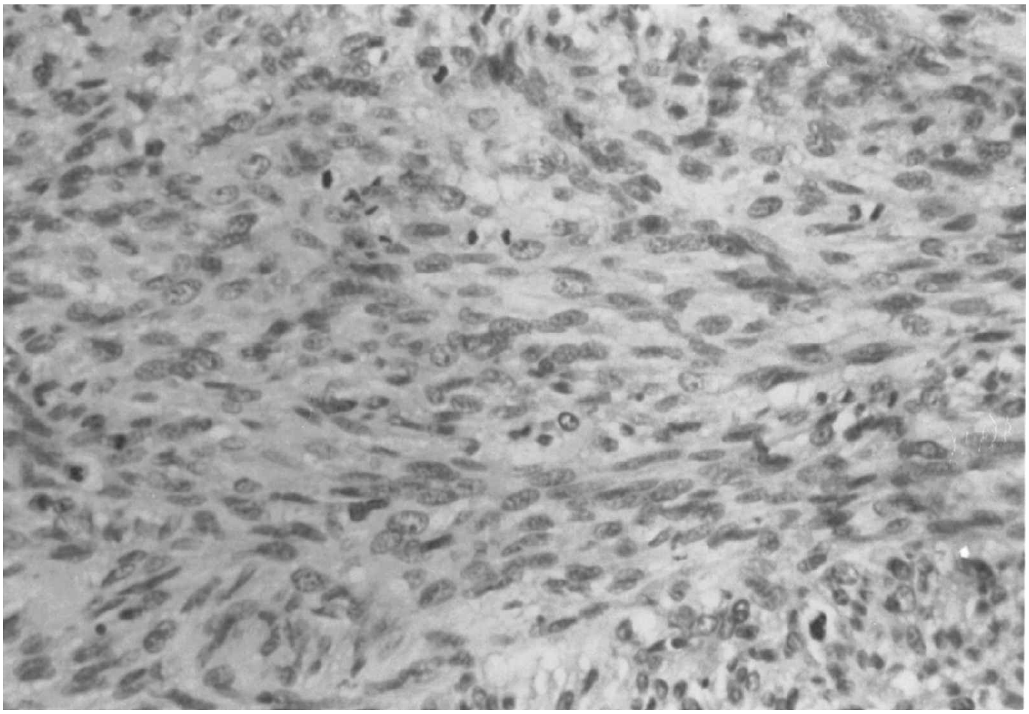

**Fig. 3.** *Histological appearance of the tumour demonstrating gross pleiomorphism of smooth muscle fibres with areas of necrosis and hyalinisation (x 300).*

**Table 1.** *Previously reported venous leiomyosarcomas of the extremities*

| Date | Author | Sex | Age (years) | Site | Size (cm) | Metastases | Follow-up (years) |
|---|---|---|---|---|---|---|---|
| 1919 | Van Ree[2] | F | 42 | Long saphenous | | | 1.4 |
| 1954 | Haug & Loesli[13] | M | 53 | Femoral | 3 | Lung | 2.4 |
| 1955 | Font & Noer[2] | M | 50 | Antecubital | 1.5 | | Unknown |
| 1955 | Johnston & Shands[14] | F | 67 | Femoral | 3 | | 0.3 |
| 1958 | de Weese *et al.*[15] | M | 54 | Femoral | 6 | | 5 |
| 1958 | Stout & Hill[16] | M | 51 | Femoral | 1 | Lung | Unknown |
| 1963 | Dorfman & Tishel[17] | M | 56 | Long saphenous | 3 | | 0.1 |
| 1964 | Christiansen[2] | F | 64 | Long saphenous | 4.5 | | 0.2 |
| 1965 | Allison[18] | F | 3 | Long saphenous | 1 | | 0.6 |
| 1966 | Sakurai *et al.*[19] | M | 54 | Femoral | 2 | | 0.6 |
| 1969 | Leu & Nipkow[2] | M | 40 | Long saphenous | 1 | | 18 |
| 1969 | Szaz *et al.*[20] | M | 68 | Long saphenous | 5.5 | Liver | 4 |
| 1973 | Hughes[21] | F | 53 | Long saphenous | 2.5 | | 0.6 |
| 1975 | Jernstrom & Gowdy[22] | M | 64 | Long saphenous | 12 | Lung | 1.2 |
| 1975 | Gross & Horton[23] | M | 46 | Long saphenous | 12 | Thyroid | 3 |
| 1977 | Stringer[24] | F | 36 | Long saphenous | 6 | Lung | 11 (D) |
| 1977 | Stringer[24] | M | 39 | Long saphenous | | | Unknown |
| 1977 | Dzinich *et al.*[25] | F | 70 | Long saphenous | | | 17 |
| 1977 | Dzinich *et al.*[25] | F | 54 | Long saphenous | | Lung | 0.9 (D) |
| 1979 | Varela-Duran[1] | M | 66 | Popliteal | | Lung | 3 |
| 1982 | Fischer *et al.*[26] | F | 66 | Long saphenous | 2 | | 4 |
| 1984 | Berlin *et al.*[3] | M | 60 | Long saphenous | 3 | Lung | 0.1 (D) |
| 1984 | Berlin *et al.*[3] | M | 42 | Axillary | 16 | Lung | 1 |
| 1984 | Berlin *et al.*[3] | M | 72 | Femoral | 7 | Lung | 4 (D) |
| 1984 | Berlin *et al.*[3] | M | 58 | Femoral | 9 | Lung | 4 (D) |
| 1984 | Berlin *et al.*[3] | F | 63 | Femoral | 8 | Lung | 2 |
| 1984 | Berlin *et al.*[3] | M | 63 | Popliteal | 18 | Lung | 0.3 (D) |
| 1986 | Leu & Makek[4] | F | 56 | Short saphenous | 8 | Lung | 2 (D) |
| 1986 | Leu & Makek[4] | M | 61 | Dorsum of hand | 2 | | 14 |
| 1987 | Humphry *et al.*[27] | M | 45 | Long saphenous | 2.5 | | |
| 1988 | Basu *et al.*[28] | F | 35 | Popliteal | 9.5 | Lung | Unknown |
| 1992 | Stallard *et al.*[29] | F | 64 | Long saphenous | 7 | | Unknown |
| 1994 | Begin *et al.*[30] | F | 75 | Dorsal pedal | 2.5 | | 5 |

*(D)—denotes dead at latest follow up.

common in both sexes — tumours of the inferior vena cava are much more common in females.[2]

Like other soft tissue sarcomas of the extremities, the lesions are frequently misdiagnosed as thrombophlebitis, lipomas, muscle hernias or lymphadenopathy. In an attempt to optimise pre-operative diagnosis and treatment, Rydholm has proposed that referral to a soft tissue tumour specialist is appropriate for masses that are larger than 5 cm, deep seated or otherwise suspicious of malignancy.[6] Such criteria resulted in an 80% pre-operative referral rate to his unit, which may confer a threefold reduction in local recurrence rates.[7] Magnetic resonance imaging is the staging modality of choice, and pre-operative needle or incisional biopsy should be performed within the specialist unit.

Size relates to prognosis, as do tumour grade, extra-compartmental spread, proximal location and deep fixation.[8] Failure to obtain a wide or radical surgical margin is associated with a higher local recurrence rate.[9,10] Metastases are usually occult at presentation, but a large proportion of patients subsequently develop macroscopic metastases, usually in the lungs. Surgical excision of isolated metastases can be advantageous.

Radiotherapy to the primary tumour site has been shown to reduce the local recurrence rate of soft tissue sarcomas.[11] There is considerable current debate about the relative merits of neo-adjuvant versus adjuvant radiotherapy.[12] Until recently, the benefits of adjuvant chemotherapy in apparently non-metastatic disease have also been disputed. A recent meta-analysis from the Sarcoma Meta-analysis Collaboration (SMAC) analysed data from 14 randomised, controlled trials investigating the value of adjuvant doxorubicin-based chemotherapy for adults with localised, resectable soft-tissue sarcoma.[13] There was no significant improvement in overall survival with such chemotherapy, but local and distal recurrence was significantly delayed — particularly in tumours of the extremities. The authors have proposed that a survival advantage might be concealed by the subsequent, non-randomised administration of doxorubicin-based chemotherapy to patients with recurrent disease; treatment-naive tumours might be expected to show a better response.

### References

1 Varela-Duran J, Oliva H, Rosai J. Vascular leiomyosarcoma: the malignant counterpart of vascular leiomyoma. *Cancer* 1979; 44:1684–91.

2 Kevorkian J, Cento D. Leiomyosarcoma of large arteries and veins. *Surgery* 1973; 73:390–400.

3 Berlin O, Angervall L, Kindholm LG, Berlin IC, Stener B. Leiomyosarcomas of venous origin in the extremities: a correlated clinical, roentgenographic and morphological study with diagnostic and surgical implications. *Cancer* 1984; 54:2147–59.

4 Leu H, Makek M. Intramural venous leiomyosarcomas. *Cancer* 1986; 57:1395–400.

5 Rydholm A. Improving the management of soft tissue sarcoma. Diagnosis and treatment should be given in specialist centres [editorial]. *Br Med J* 1998; 317:93–4.

6 Gustafson P, Dreinoefer K, Rydholm A. Soft tissue sarcoma should be treated at a tumor center. A comparison of quality of surgery in 375 patients. *Acta Orthop Scand* 1994; 65:47–59.

7 Vraa S, Keller J, Nielsen OS, Sneppen O, Jurik AG, Jensen OM. Prognostic factors in soft tissue sarcomas: the Aarhus experience. *Eur J Cancer* 1998; 34:1876–82.

8 Enneking W, Spanier S, Goodman M. A system for the surgical staging of musculoskeletal sarcoma. *Clin Orthop* 1980; 153:106–20.

9 Rydholm A. Surgical margins for soft tissue sarcoma. *Acta Orthop Scand* 1997; 273:68.

10 Suit H, Mankin HJ, Wood WC, Proppe KH. Preoperative, intraoperative and postoperative radiation in the treatment of primary soft tissue sarcoma. *Cancer* 1985; 55:2659–67.

11 Robinson M, Keus RB, Sharha D, Harrison LB. Is pre-operative radiotherapy superior to post-operative radiotherapy in the treatment of soft tissue sarcoma? *Eur J Cancer* 1998; 34:1309–16.

12 Sarcoma Meta-analysis Collaboration. Adjuvant chemotherapy for localised resectable soft tissue sarcoma in adults: meta-analysis of individual data. *Lancet* 1997; 350:1647–54.

13 Haug W, Loesli E. Primary leiomyosarcoma within the femoral vein. *Cancer* 1954; 7:159.

14 Johnston J, Shands W. Primary leiomyosarcoma of the femoral vein. *Surgery* 1955; 38:410.

15 De Weese J, Banner HB, Mahoney EB, Rob CG. Leiomyosarcoma of the great saphenous vein with preoperative localisation by phlebography. *Ann Surg* 1958; 148:859–61

16 Stout A, Hill W. Leiomyosarcomas of the superficial soft tissues. *Cancer* 1958; 11:844.

17 Dorfman H, Tishel E. Leiomyosarcoma of the greater saphenous vein. *Am J Clin Pathol* 1963; 39:73–7.

18 Allison M. Leiomyosarcoma of the femoral vein. *Clin Pediatr* 1965; 4:28–31.

19 Sakurai O, Toda A, Morimoto K. Primary leiomyosarcoma within the femoral vein. *Clin Orthop* 1966; 44:197.

20 Szaz I, Barr R, Scobie TK. Leiomyosarcoma arising from the veins: two cases and a review of the literature on venous neoplasms. *Can J Surg* 1969; 12:415.

21 Hughes S. A case of leiomyosarcoma of the long saphenous vein. *Vasc Surg* 1973; 7:71–3.

22 Jernstrom P, Gowdy R. Leiomyosarcoma of the long saphenous vein. *Am J Clin Pathol* 1975; 63:25–31.

23 Gross E, Horton M. Leiomyosarcoma of the saphenous vein. *J Pathol* 1975; 116:37–41.

24 Stringer B. Leiomyosarcoma of artery and vein. *Am J Surg* 1977; 134:90–4.

25 Dzinich C, Gloviczki P, Van Heerden J, *et al.* Primary venous leiomyosarcoma: a rare but lethal disease. *J Vasc Surg* 1992; 15:595–603.

26 Fischer M Gelb A, Nussbaum M, Haveson S, Ghali V. Primary smooth muscle tumours of venous origin. *Ann Surg* 1982; 196:720–4.

27 Humphry M, Neff J, Lin F, Krishnan L. Leiomyosarcoma of the saphenous vein: a case report and review of the literature. *J Bone Joint Surg (Am)* 1987; 69:282.

28 Basu S, Scott T, Wilmshurst C, MacEachern A, Clyne C. Leiomyosarcomata of the popliteal vessels: rare primary tumours. *Eur J Vasc Surg* 1988; 2:423–5.

29 Stallard D, Sundaram M, Johnson F, Janney C. Case report 747: leiomyosarcoma of the great saphenous vein. *Skeletal Radiol* 1992; 21:399–401.

30 Begin L, Guy P, Mitmaker B. Intramural leiomyosarcoma of the dorsal pedal vein: clinical mimicry of ganglion. *Foot Ankle* 1994; 15:48–51.
